# Public Perceptions of the Emerging Human Monkeypox Disease and Vaccination in Riyadh, Saudi Arabia: A Cross-Sectional Study

**DOI:** 10.3390/vaccines10091534

**Published:** 2022-09-15

**Authors:** Sultan Ayoub Meo, Thamir Al-Khlaiwi, Ziyad Fahad Aljofan, Aued Iaed Alanazi, Anusha Sultan Meo

**Affiliations:** 1 Department of Physiology, College of Medicine, King Saud University, Riyadh 11461, Saudi Arabia; 2 College of Medicine, King Saud University, Riyadh 11461, Saudi Arabia

**Keywords:** human monkeypox, disease, knowledge, Saudi Arabia

## Abstract

The human monkeypox disease is caused by the monkeypox virus (MPXV), which is a zoonotic disease. In the year 2022, the prevalence of monkeypox cases swiftly increased worldwide and the disease has now been declared a global public health emergency. The present study aimed to assess the public’s perceptions and knowledge of and attitudes toward monkeypox in Riyadh, Saudi Arabia. This questionnaire-based cross-sectional study was conducted from 15 May to 15 July 2022. The participants’ perceptions, knowledge, and attitudes were collected via a 28-item-based questionnaire survey. The survey was based on 1020 participants (554 (54.3%) were females, and 466 (45.7%) were males). The results reveal that out of 1020 participants, 799 (78.3%) respondents believed that monkeypox disease has developed into a pandemic situation, and 798 (78.2%) suggested that the disease is most common in Western and Central Africa. Further analysis shows that 692 (67.8%) respondents agreed that monkeypox cases are increasing worldwide, 798 (21.8%) believed that monkeypox is commonly transmitted through direct contact, and 545 (53.4%) of respondents reported that it is easily transmitted from human to human. Moreover, 693 (67.9%) participants mentioned that monkeypox disease is spreading more widely as people travel from one country to another, while 807 (79.1%) participants were aware that smallpox and monkeypox have similar clinical features. Furthermore, the majority of participants (*p* = 0.033) agreed that health officials should start a vaccination campaign to combat monkeypox. Regarding preventive measures and vaccination campaigns, 641 (62.8%) participants suggested that health officials should take public preventive measures and 446 (43.7%) recommended that health officials start vaccination campaigns against monkeypox. The knowledge of human monkeypox among the general population in Riyadh, Saudi Arabia was satisfactory for all ages, genders, levels of education, and economic groups. Moreover, the majority of participants proposed adopting preventive measures and starting a vaccination campaign to combat monkeypox disease. The knowledge of monkeypox in the public domain is a key factor to improve the public‘s capacity to minimize the disease burden and fight against viral infectious diseases at regional and global levels.

## 1. Introduction

The human monkeypox disease, known simply as monkeypox disease, is caused by a monkeypox virus (MPXV) and is a zoonotic infectious disease, frequently found in African countries [1,2]. The MPXV belongs to the “*genus Orthopoxvirus, subfamily Chordopoxvirinae and family Poxviridae*”. The genomes of these viruses are ≈200 kb long, with replication characteristics, and are involved in host range determination and pathogenesis [3,4,5].

The possible pathogenesis and transmission of MPXV are animal–animal, animal-human, and human-human transmission. Direct or indirect contact with bodily fluids, respiratory droplets, the skin lesions of an infected person, and patients’ contaminated possessions, bedding, clothing, and environment have been associated with inter-human transmission [4,6].

The transmission of disease may be due to close physical, skin-to-skin, or face-to-face interaction [7,8]. MPXV infection can also be transmitted through bites or scratches from infected animals, and the contamination of raw meat [9]. Moreover, rodents and squirrels may also play a role in the transmission of MPXV to humans [9].

The MPXV was first found in 1958 among a group of monkeys housed in a research institute in Copenhagen, Denmark [10]. About 12 years later, in September 1970, the MPXV was identified for the first time in humans in the Democratic Republic of Congo [11,12]. In the new millennium, in the year 2003, the first case of the MPX disease was reported from endemic to non-endemic countries [13,14].

The World Health Organization has stated that MXPV disease is a global emergency [15]. This year, from 1 January 2022 to 19 August 2022, MPXV has swiftly spread from non-endemic to endemic regions [16], involving 94 countries and infecting 41,358 people; 387 cases were reported from seven endemic African countries and 40,971 cases in 87 non-endemic countries in Europe, America, Australia, and the Asian continent [14].

Currently, there is no specific approved vaccine for MPXV. However, vaccination against the smallpox virus provided cross-protection against MPXV [17]. There are three generations of vaccines used against the smallpox virus [18]. The first-generation vaccine was used against smallpox until 2008. This vaccine was highly effective in preventing smallpox and played a vital role in the eradication of smallpox all around the world. In 1980, the WHO declared the eradication of smallpox, and this vaccine was discontinued [7]. The second-generation vaccine, the live attenuated tissue culture-derived vaccinia virus vaccine has been used for populations who might be at high risk for orthopoxvirus. The third-generation vaccine, the modified vaccinia Ankara-Bavarian Nordic (MVA-BN), was approved for human use in Canada and Europe [7].

In the present situation of global emergency, public awareness about monkeypox disease is vital to educating the people to fight against such infectious diseases. Therefore, the present study aimed to assess the public perceptions and knowledge of monkeypox in Riyadh, Saudi Arabia.

## 2. Subjects and Methods

### 2.1. Study Design and Settings

This questionnaire-based cross-sectional survey was steered in the “Department of Physiology, College of Medicine, King Saud University, Riyadh, Saudi Arabia”, from 15 May to 15 July 2022.

### 2.2. Study Area Demographics

The total population of Saudi Arabia is around 35.8 million; the male population is 20.70 million and the female population is 15.14 million, with a median age of 32.4 years. Riyadh is the largest city and the capital of Saudi Arabia. The city consists of five regions with a total population of about 7.54 million people, comprising 57% males and 43% females. More Saudi women are studying in universities than men; there are about 551,000 women and 513,000 men studying for bachelor’s degrees in universities.

### 2.3. Study Sample Size

The targeted study participants were Saudi and non-Saudi male and female residents in the capital city of Riyadh, Saudi Arabia. A power formula was used to calculate the sample size, based on a 50% population proportion, a 95% confidence interval, and a 5% margin of error. For this study, a sample size of about 800 people was required, but the number of participants who responded and were included in the study analysis was 1020.

### 2.4. Study Survey Procedure and Instrument

The study participants were invited to join the questionnaire survey using social media platforms (via WhatsApp and emails). The study objectives and a polite request for their consent and voluntary participation were presented at the beginning of the questionnaire. The study variables were socio-demographic characteristics, age, gender, occupation, level of education, socioeconomic status, and allied questions about knowledge, attitude, and perceptions regarding monkeypox disease. The study was based on a well-designed questionnaire [19] issued to the targeted population of Riyadh, Saudi Arabia. The survey participants represent the entire Riyadh region of Saudi Arabia.

After receiving permission, the questionnaire [19] was slightly modified and used for the data collection. The reliability and technical issues of the questionnaire were tested among 10 participants, and their feedback was taken in the pilot survey test. After ethical approval, the questionnaire was distributed online through email, Google, and social media platforms in Riyadh, Saudi Arabia. The questionnaire was distrusted by 1300 participants, 1020 (78.46) participants responded to the survey, and 280 (21.53%) did not respond to the survey. Among the 1020 participants, 554 (54.3%) were females, and 466 (45.7%) were males.

The names of the participants were not collected to maintain confidentiality. The invitations to participate in the online survey were distributed by the social media program WhatsApp and e-mails. To achieve a better response, one reminder was also sent as a follow-after the initial message. An introductory page consisted of information on the research objectives, demographic information, and the expected benefits. The survey was estimated to take about 10 min to complete. The duplication distribution of the questionnaire was checked, and the raw data were extracted and imported for analysis. The questionnaire consisted of 28 questions to assess the knowledge, attitudes, and perceptions regarding monkeypox. The questionnaire was developed in the national language of Arabic, and also in the English language.

### 2.5. Ethical Considerations

An introductory page was provided, informing the participants that they could exit the survey at any point, and before enrolling, they were asked to provide their consent to participate. This study was approved by the Ethics Committee Institutional Review Board (IRB), College of Medicine Research Centre, King Saud University, Riyadh, Saudi Arabia (Ref: 22/0466/IRB).

### 2.6. Statistical Analysis

The results were examined using the SPSS software, version 26.0 for Mac. The demographical variables of age, gender, occupation, education, and socioeconomic status were reported, using frequency and percentage. The response score was reported using mean and standard deviation. The comparisons between the variables were analyzed using independent sample *t*-tests, ANOVA, and chi-squared tests. A *p*-value of <0.05 was considered significant.

## 3. Results

### 3.1. Demographic Profile of Respondents

Table 1 represents the demographic profiles of the respondents. The results indicate that the majority of respondents were in the age group of 21–30 years old (558, 54.7%), followed by 15–20 years old (388, 38.0%); and 554 (54.3%) were female and 466 (45.7%) were male. Regarding the education level of the respondents, of the maximum number of participants, 608 (59.6%) had bachelor’s degrees, while 341 (33.4%) had middle- or high-school qualifications, 38 (3.7%) had master’s degrees, and 6 (0.6%) had a Ph.D. Additionally, the results indicate that most of the respondents (483, 47.4%) were from the central region and 117 (11.5%) were from the eastern region of the capital city of Riyadh. In terms of the professional level of the participants, most of them were students (644, 63.1%), followed by 79 (7.7%) who were working in the private sector, 47 (4.6%) had government jobs, and 33 (3.2%) were health practitioners. The majority of the respondents were single (932, 91.4%), while 69 (6.8%) were married and 18 (1.8%) were divorced. Their socio-economic status shows that 596 (58.4%) respondents had a monthly income of less than SAR 3000, followed by 81 (7.9%) who were earning SAR 9000 or more (Table 1).

### 3.2. The Respondents’ Awareness of Monkeypox

The respondents were presented with different statements to assess their level of understanding and information about monkeypox. The results reveal that 799 (78.3%) respondents believed that monkeypox disease has become a pandemic, 798 (78.2%) believed that this disease is most common in Western and Central Africa, and 692 (67.8%) agreed that monkeypox cases are increasing worldwide. Upon further inquiry, 798 (21.8%) believed that monkeypox disease is commonly transmitted through direct contact, and 545 (53.4%) respondents mentioned that monkeypox disease is easily transmitted from human to human. As per the viewpoint of 693 (67.9%) people, monkeypox disease is widely spreading as people travel from one country to another. Moreover, 807 (79.1%) respondents were aware that smallpox and monkeypox have similar symptoms, while half of the respondents (491, 48.1%) were aware of the clinical symptoms of monkeypox disease and a large number of respondents (889, 87.2%) knew that a skin rash is the main symptom of the disease. There is a distinction between smallpox and monkeypox, and 585 (57.4%) respondents knew the other clinical feature of monkeypox disease. About 728 (71.4%) participants knew that smallpox vaccinations can be used to protect against monkeypox, and 278 (27.3%) respondents claimed to know about the complications of monkeypox (Table 2).

**Figure 1:**
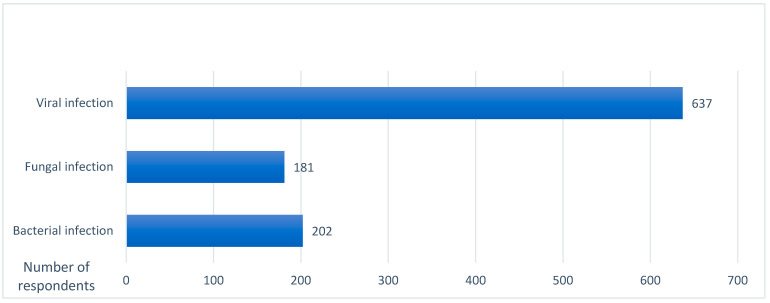
Public perceptions regarding the types of monkeypox disease (viral, fungal, bacterial).

Furthermore, the respondents were questioned about their opinion regarding what kind of disease monkeypox is. Interestingly, 202 (19.8%) thought that it is a bacterial infection, 181 (17.7%) considered it a fungal infection, and 637 (62.5%) thought that it is a viral infection (Table 3, Figure 1).

### 3.3. Preventive and Recommendation Measures

**Figure 2:**
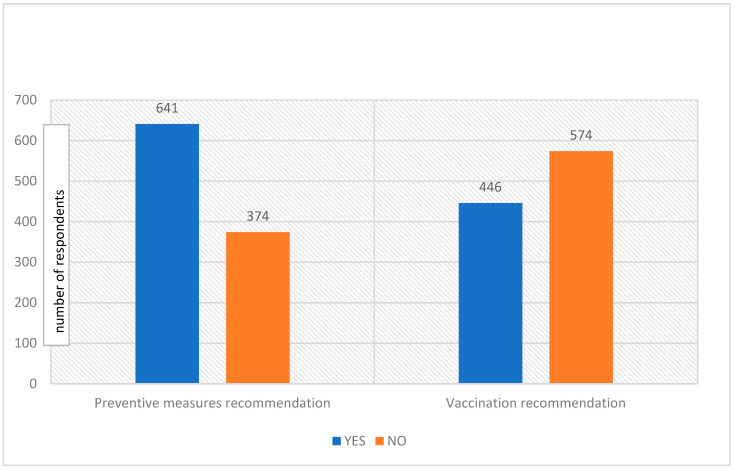
Public recommendations, preventive measures, and vaccination campaigns against monkeypox disease.

This section reports the precautions taken by the respondents to avoid getting infected with monkeypox (Table 4, Figure 2). First of all, the respondents were asked if they were afraid of contracting monkeypox; in response to this query, 412 (40.4%) answered yes. Moreover, 141 (13.8%) respondents were afraid of visiting family or friends, due to the chance of contracting monkeypox, while the majority (879, 86.2%) would not stop visiting anyone. Furthermore, 399 (39.1%) respondents would not travel to other countries as they were afraid of contracting monkeypox disease, and 228 (22.4%) respondents suggested taking hygienic preventive measures to prevent this disease.

### 3.4. Perceptions of a Vaccination Campaign against Monkeypox

The Chi-squared test has been applied to check the association between the demographic variables and the recommendation of the respondents that health officials should start a vaccination campaign. The results reveal that there was no relationship between recommending that health officials should start the vaccination campaign and the respondents’ age (*p* = 0.110), gender (*p* = 0.216), occupation (*p* = 0.560) and socioeconomic status (*p* = 0.672). However, there is a statistically significant association between the recommendation of health officials to start vaccination campaigns and education level (*p* = 0.033) Table 5.

### 3.5. Preventive Measures and Recommendations for Monkeypox Vaccination

The data were further analyzed between the preventive measures taken and the recommendation for monkeypox vaccination. The results indicate that the respondents were afraid due to the risk of monkeypox disease, and the participants were more likely to recommend vaccination as they do not want to suffer from the virus and the vaccine is essential for the prevention of the disease (*p* < 0.01). The results reveal that 63.3% of respondents were afraid of the virus and that they would recommend that health officials start a vaccination program for the disease. Moreover, if people are taking precautions for the hygienic prevention of monkeypox, they would prefer to receive the vaccine as well, as hygiene is important but is not the only preventive measure against the virus. Furthermore, 68.9% of respondents took preventive measures and recommended that health officials start a vaccination program. The two conditions were associated with each other (*p* < 0.01). Additionally, monkeypox disease has caused a fear of visiting family and friends so that people could protect themselves from the virus; it was observed that 77.3% of respondents have limited their visits to family and friends as a result and they recommend the vaccination for MPXV; these two situations were linked with each other (*p* < 0.01). Monkeypox fears have also limited their visit to other countries; 57.6% of respondents agreed that they are not travelling to other countries because they are fearful of catching the virus and recommend that health officials should start a vaccination program against monkeypox disease and that these two variables have an association with each other (*p* < 0.01) (Table 6).

## 4. Discussion

Since early May 2022, the human monkeypox outbreak across many countries has raised concerns about a possible change in the pattern of monkeypox transmission, and that the disease now poses a greater global threat [15]. The transmission of monkeypox diseases is not only limited to the close contract but it can also be transmitted through respiratory droplets, direct or indirect contact with bodily fluids, certain possessions, the skin lesions of an infected person, and a contaminated patient’s environment [4,6].

The present study findings suggest that the general population in Riyadh, Saudi Arabia has a satisfactory level of knowledge about the human monkeypox disease. The majority of participants proposed adopting preventive measures and initiating a vaccination campaign to eradicate monkeypox disease at regional and global levels. The present study findings reflect high levels of endorsement of public perceptions about the emerging monkeypox threat. Monkeypox disease was declared a global emergency on 23 July 2022 by the WHO [16] and alarm bells began to ring worldwide, as a reminder of the similar situation that arose immediately before the spread of the severe acute respiratory syndrome coronavirus 2 (SARS-CoV-2), also known as COVID-19, which was declared a global pandemic in March 2020 [20].

Meo et al. (2022) [15] reported that the number of cases of monkeypox is now surging drastically. From what started as an endemic zoonotic disease that was restricted to Central and West African countries, it was not until earlier this year that the number of cases began to climb in countries where monkeypox had never existed before, indicating that the disease is now becoming a global health concern. Monkeypox has now spread to around 94 states across the world. With an emergency situation declared, the international community must immediately act to prepare for a possible pandemic, so that this time, the global healthcare system does not lack sufficient supplies and is not taken by surprise, as it was in 2020 when the COVID-19 pandemic first spread [20].

Harapan et al. (2020) [19] reported that knowledge regarding monkeypox among a group of general practitioners in Indonesia was low, although knowledge about monkeypox is essential to enhance the profession’s capacity to respond to human monkeypox prevalence.

In another study, Sallam et al. (2022) [21] reported that knowledge regarding the emerging monkeypox disease among 615 university students in Jordanian health schools was unsatisfactory. About 26.0% of the respondents knew that vaccination could help to prevent monkeypox. Age was associated with better human monkeypox (HMPX) knowledge. Our study, with a sample size of 1020, had satisfactory results, with more than half of the respondents answering most questions appropriately. About 71.4% of participants knew that smallpox vaccinations can be used to protect against monkeypox. The knowledge of human monkeypox among the general population in Riyadh, Saudi Arabia, was satisfactory for all ages, genders, levels of education, and economic groups.

Riccò et al. (2022) [22] conducted a study in Italy that involved 566 participants and reported that the knowledge status of its participants was quite unsatisfactory, with substantial knowledge gaps on all aspects of monkeypox. In our study, 71.4% of respondents were aware of the smallpox vaccine’s role in protecting against monkeypox, and only 58.6% of respondents in the Italian study were somewhat in favour of implementing variola vaccinations to prevent monkeypox.

Nonetheless, in terms of knowledge about monkeypox and a positive attitude toward future monkeypox vaccines, it is essential to educate communities about such emerging viral diseases. An important tool for dealing with a health emergency crisis is to ensure the education of the masses regarding how to approach, deal with, and protect oneself if nearby people become infected. Providing essential knowledge ensures that the public takes the necessary precautions themselves and relieves the burden on the healthcare authorities by limiting the spread and surging of the disease. These steps must be taken in adequate time before the onset of its general spread; analyzing the pre-existing knowledge level of the public is the most important way to combat infectious diseases.

### 4.1. Study Strengths and Limitations

The strength of this study is that this is among the first studies to correlate the public’s knowledge of the emerging monkeypox outbreak in Riyadh, Saudi Arabia. The results of this study may be helpful in providing awareness programs to further improve the public’s knowledge of virus emergence and diseases. The sample size was suitable for representing the general public’s perceptions. The limitation of this study was that the data were collected only from the capital city of the country; it would be more appropriate to collect additional data from other cities in the country.

### 4.2. Recommendations

It is essential to educate the public and provide timely advice to increase public awareness of monkeypox disease through lectures, seminars, and the involvement of electronic and print media. The implementation of a “One Health approach”, a multidisciplinary and multi-sectoral, collaborative, and transdisciplinary approach at the inland, regional, national, and global levels, with one objective of achieving ideal health outcomes and identifying the interconnection between people, animals, plants, and the environment.

Moreover, the early diagnosis of patients, rapid notification to health authorities about suspected cases of MPXV, and the implementation of public intervention measures are decisive in eradicating the disease. Furthermore, the smallpox vaccine campaign and the development of antiviral drugs to treat this neglected tropical disease are highly recommended.

## 5. Conclusions

The knowledge of human monkeypox among the general population in Riyadh, Saudi Arabia was satisfactory for all ages, genders, levels of education, and economic groups. The majority of participants showed a positive attitude and proposed adopting preventive measures and starting a vaccination campaign to combat the spread of monkeypox disease. The knowledge of monkeypox disease among the public is a key factor to improve the public capacity to minimize the disease burden and fight against viral infectious diseases at regional and global levels.

## Figures and Tables

**Table 1:** Demographic profile of the study participants.

Variable	Value	Frequency	Percentage
Age	15–20 years	388	38.0
	21–30 years	558	54.7
	31–40 years	58	5.7
	41–50 years	15	1.5
	70 years and above	1	0.1
Gender	Female	554	54.3
	Male	466	45.7
Educational level	Bachelor’s degree	608	59.6
	Elementary	1	0.1
	Master’s degree	38	3.7
	Middle/High school	341	33.4
	Other (Diploma)	25	2.5
	Ph.D./Fellowship	6	0.6
Region	Central region	483	47.4
	Eastern region	117	11.5
	Northern region	65	6.4
	Southern region	3	0.3
	Western region	352	34.5
Occupation	Governmental job (ex. teachers)	47	4.6
	Health practitioner	33	3.2
	Military(police)	12	1.2
	Private sector	79	7.7
	Student	644	63.1
	Unemployed	205	20.1
Marital status	Divorced	18	1.8
	Married	69	6.8
	Single	932	91.4
	Widowed	1	0.1
Socioeconomic status (monthly)	<3000 SAR	596	58.4
	10,000 or more SAR	1	0.1
	3000–6000 SAR	78	7.6
	6000–less than 9000 SAR	46	4.5
	9000 or more SAR	81	7.9

**Table 2:** Participants’ awareness of the monkeypox virus and disease.

Statements	Yes (*n* and (%))	No (*n* and (%))
Do you believe that monkeypox disease has become a pandemic?	799 (78.3)	221 (21.7)
Do you know that monkeypox is common in Western and Central Africa?	798 (78.2)	222 (21.8)
Do you know that monkeypox cases are increasing worldwide?	692 (67.8)	328 (32.2)
Are monkeypox cases present in Saudi Arabia?	914 (89.6)	106 (10.4)
Do you know monkeypox is commonly transmitted through direct contact?	798 (78.2)	222 (21.8)
Do you know monkeypox is easily transmitted from human to human?	545 (53.4)	475 (46.6)
Do you know that travelling to other countries is the main cause of spreading monkeypox disease?	693 (67.9)	327 (32.1)
Do you know that monkeypox and smallpox have similar signs and symptoms?	807 (79.1)	213 (20.9)
Do you know the clinical symptoms of monkeypox disease?	491 (48.1)	529 (51.9)
Do you know that a skin manifestation (skin rash) is the main symptom of monkeypox disease?	889 (87.2)	131 (12.8)
Do you know that lymphadenopathy (swollen lymph nodes) is one clinical feature that could be used to differentiate between monkeypox and smallpox?	585 (57.4)	435 (42.6)
Do you know that the smallpox vaccination protects against the human monkeypox virus?	728 (71.4)	292 (28.6)
Do you know the complications of monkeypox disease?	278 (27.3)	742 (72.7)

**Table 3:** Public perceptions regarding the types of monkeypox disease (bacterial, fungal, viral).

	Frequency	Percentage
Bacterial infection	202	19.8
Fungal infection	181	17.7
Viral infection	637	62.5

**Table 4:** Preventive and recommended measures against monkeypox disease.

Questions	Yes (*n* and (%))	No (*n* and (%))
Are you afraid of catching monkeypox disease?	412 (40.4)	608 (59.6)
Are you afraid to visit any family members or friends due to monkeypox disease?	141 (13.8)	879 (86.2)
Are you afraid to travel to any country due to monkeypox disease?	399 (39.1)	621 (60.9)
Are you taking more hygienic preventive measures due to monkeypox disease?	228 (22.4)	792 (77.6)
**Recommendations**		
Do you recommend that health officials start a vaccination campaign against monkeypox disease?	446 (43.7)	574 (56.3)
Do you recommend that health officials should take public preventive measures?	641 (62.8)	374 (37.2)

**Table 5:** Inferential statistics regarding recommendations about the vaccination campaign against monkeypox, in association with the sociodemographics of the respondents.

Variables	Do You Recommend Health Officials to Start a Vaccination Campaign against Monkeypox?		
	Yes (*n* and (%))	No (*n* and (%))	*p*-Value
**Age**			0.110
15–20 years	185 (47.7)	203 (52.3)	
21–30 years	235 (42.1)	323 (57.9)	
31–40 years	20 (34.5)	38 (55.5)	
41–50 years	5 (33.3)	10 (66.7)	
70 years and above	1 (100.0)	0 (0.0)	
**Gender**			0.216
Male	194 (41.6)	272 (58.4)	
Female	252 (45.5)	302 (54.5)	
**Education level**			0.033
Bachelor’s	246 (40.5)	362 (59.5)	
Elementary	0 (0.0)	1 (100.0)	
Master’s	16 (42.1)	22 (57.9)	
Middle/High school	168 (49.3)	173 (50.7)	
Other (Diploma)	11 (44.0)	14 (56.0)	
Ph.D./Fellowship	5 (83.3)	1 (16.7)	
**Occupation**			0.560
Governmental job (ex. teachers)	21 (44.7)	26 (55.3)	
Health practitioner	11 (33.3)	22 (66.7)	
Military(police)	3 (25.0)	9 (75.0)	
Private sector	32 (40.5)	47 (59.5)	
Student	290 (45.0)	354 (55.0)	
Unemployed	89 (43.4)	116 (56.6)	
**Socioeconomic status**			0.672
<3000 SAR	262 (44.0)	334 (56.0)	
10,000 or more SAR	1 (100)	0 (0.0)	
3000–6000 SAR	39 (50.0)	39 (50.0)	
6000–less than 9000 SAR	18 (39.1)	28 (60.9)	
9000 or more SAR	33 (40.7)	48 (59.3)	

**Table 6:** Public perceptions about the preventive measures and recommendations for monkeypox vaccination.

Variables	Do You Recommend that Health Officials Start the Vaccination Campaign against Monkeypox Disease?		
	No (*n* (%))	Yes (*n* (%))	*p*-Value
Are you afraid of monkeypox disease?			
No	423 (69.6)	185 (30.4)	0.000
Yes	151 (36.7)	261 (63.3)	
Are you taking more hygienic preventive measures due to monkeypox disease?			0.000
No	503 (63.5)	289 (36.5)	
Yes	71 (31.3)	157 (68.9)	
Are you afraid to visit any family members or friends due to monkeypox disease?			0.000
No	542 (61.7)	337 (38.3)	
Yes	32 (22.7)	109 (77.3)	
Are you afraid to travel to any country due to monkeypox disease?			0.000
No	405 (65.2)	216 (34.8)	
Yes	169 (42.4)	230 (57.6)	

## Data Availability

May be provided on reasonable request to the corresponding author.
